# Pharmacy Deserts and Pharmacies’ Roles Post-Extreme Weather and Climate Events in the United States: A Scoping Review

**DOI:** 10.1177/21501319231186497

**Published:** 2023-07-11

**Authors:** Harpreet Sahota, Samantha Guzman, Lissette Tordera, Michelle Chan, Jennifer Cocohoba, Parya Saberi

**Affiliations:** 1 University of California, San Francisco, San Francisco, CA, USA

**Keywords:** pharmacy desert, pharmacist, natural disaster, extreme weather events, health disparities

## Abstract

**Background:**

The effects of climate change are seen with a rise of extreme weather and climate events (EWCEs) which lead to the closures of many healthcare facilities, such as community pharmacies. Pharmacists in community pharmacies are seen as the most accessible healthcare professional to the public and are responsible for the continued delivery of care to patients. However, amid closures due to EWCEs and the emergence of pharmacy deserts, there is decreased access to pharmacies and a disruption of care.

**Objective:**

It is important to address the preparedness and accessibility of pharmacies post-EWCEs to guide future research and policy. Additionally, to tackle health disparities that arise due to pharmacy deserts, the populations most affected by a decreased access to pharmacies should be identified. We conducted a scoping review to assess the preparedness and accessibility of pharmacies post-EWCEs and to identify populations most affected by pharmacy deserts.

**Methods:**

We searched PubMed, Embase, and Web of Science from January 1, 2012 to September 30, 2022 and included all English-language, peer-reviewed primary literature that examined the preparedness and accessibility of community pharmacies in the United States post-EWCEs and addressed disparities within pharmacy deserts. Studies meeting these criteria were screened of their titles and abstracts by the first author and discrepancies were resolved with co-authors. We used Covidence for data extraction.

**Results:**

A total of 472 studies were identified (196 duplicates removed) and after screening, 53 studies were assessed for eligibility. The results of included publications (N = 26) showed that pharmacists and pharmacies are not equipped with the necessary emergency protocols which could lead to decreased access of pharmacies in the wake of EWCEs. Pharmacy deserts disproportionately affect residents living in rural, lower income, and Black/African American and Hispanic/Latino neighborhoods. The lack of preparedness of pharmacies post-EWCEs could worsen medication access.

**Conclusion:**

This scoping review addresses challenges impacting pharmacies and patients post-EWCEs and within pharmacy deserts. In times of increased need, these challenges implicate the well-being of communities affected by EWCEs by breaking the continuum of care and access to medications. Here we offer suggestions for future research and directions for policy change.

## Introduction

In the United States, pharmacists in community pharmacy settings are often at the forefront of healthcare delivery as they are the most accessible and frequently visited members of the healthcare team.^1^ Community pharmacies, in particular, play a critical role in communities by servings as an important point of medication access and health services such as vaccinations. Analogous to food deserts, pharmacy deserts are locations characterized by poor access to pharmacies, often defined as neighborhoods where the average distance to the nearest pharmacy was 1.0 mile or more, which may be a contributing factor to health disparities among populations living in these areas.^2,3^ Pharmacy deserts have been linked to decreased access to medications and other pharmacy offered services. Due to these potential effects, it is important to study factors that may impact the emergence and trends of pharmacy deserts.^3^ Equally important is to establish what populations are affected by pharmacy deserts and to strategize ways to improve health equity.

Within the last 5 years, the world has experienced a sharp increase in the frequency and severity of extreme weather and climate events (EWCEs) including hurricanes, wildfires, floods, and droughts. The continued delivery of healthcare during and post-EWCEs is crucial and challenging. Medication continuity is critical in treating patients with chronic conditions, given that medication adherence is necessary to reduce hospitalizations and sequelae associated with these conditions.^4^ Pharmacy deserts are particularly vulnerable, given that geographic areas with already limited access to medications may also be areas that are strongly impacted by EWCEs. Although EWCEs are increasingly inevitable, their short- and long-term effects on pharmacies and continued delivery of care to patients can be diminished by preparing pharmacists and pharmacies ahead of time. Thus, it is important to assess the preparedness and accessibility of pharmacies during and after EWCEs to better prepare pharmacies for future EWCEs and reduce the impact on patients.

Few studies have examined the preparedness and accessibility of pharmacists and pharmacies post-EWCEs. Thus, we conducted a scoping review to explore these topics as well as research related to pharmacy deserts. Studying these topics together allows us to recognize the level of preparedness of pharmacies, the barriers patients face when accessing pharmacies, and the populations most vulnerable which can help guide future research to investigate mitigation strategies and direct policy.

## Methods

### Search Strategy

This scoping review was conducted using the Preferred Reporting Items for Systematic Reviews and Meta-Analysis extension for Scoping Reviews (PRISMA-ScR) framework. To focus on the emergent consequences of EWCEs and pharmacy deserts, we used a timeframe of 10 years and included literature published between January 1, 2012 to September 30, 2022. We searched PubMed, Embase, and Web of Science on September 30, 2022 to identify papers that met these inclusion criteria. The search terms used were based on the keywords “pharmacy” and “deserts” and “natural disasters,” with MeSH terms adapted for the requirements of each database. Search terms were tested to ensure the identification of appropriate papers. The search strategy for PubMed was: (pharmacy[tiab] OR pharmacies[tiab] OR prescription[tiab] OR prescriptions[tiab]) AND (desert* OR earthquake* OR hurricane* OR tornado* OR tsunami* OR typhoon* OR wildfire* OR cyclone*). We hand-searched articles that were cited in included papers to identify further potential publications that met inclusion criteria.

### Screening Process

**Figure 1.**
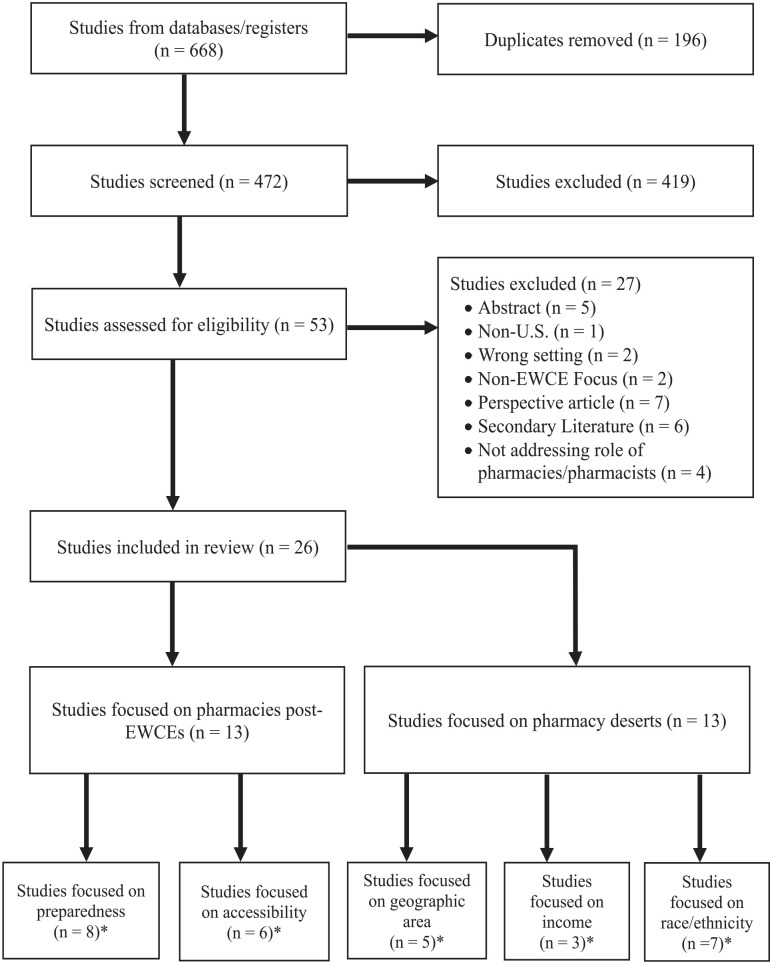
PRISMA-ScR (preferred reporting items for systematic reviews and meta-analyses extension for scoping reviews) flowchart: flow diagram of study selection. *Studies focusing on multiple areas.

References from all 3 databases were imported into Covidence where duplicates were removed, and the remaining papers were screened using the inclusion criteria. The first author reviewed the titles and abstracts of all references and discrepancies were resolved in discussion with 1 co-author. After the initial screening, we reviewed and screened the full texts of the remaining papers. Secondary literature, studies not focusing on an EWCE, not set in the United States, or not addressing the role of pharmacists or pharmacies were excluded (Figure 1). Studies that focused on the recovery efforts and aftermath of pharmacies in the United States after EWCEs were grouped together into “pharmacies post-EWCEs.” Studies that focused on the geographic accessibility of pharmacies and pharmacy services were grouped together into “pharmacy deserts.”

### Data Charting

The final papers then underwent data extraction to organize information and sort key findings through Covidence software. We extracted information such as aims, outcomes, study design (e.g., cohort or cross-sectional), geographic location (West, Midwest, South, Northeast, U.S. Virgin Islands, Puerto Rico), study population, and type of EWCE (e.g., wildfire, hurricane), if applicable. After using Covidence to extract this information, the primary author transferred the study characteristics into a Microsoft Excel spreadsheet where studies were grouped by their primary focus (pharmacy deserts or pharmacies post-EWCEs). We used descriptive statistics to analyze the data in Microsoft Excel.

## Results

A total of 472 studies were identified from the 3 databases (196 duplicates removed) and 26 studies met the eligibility criteria (Figure 1). Of the studies included, 13 (50%) examined the role of pharmacies post-EWCEs and 13 (50%) focused on pharmacy deserts (Table 1). Included studies were mostly cross-sectional (92%) or cohort studies (8%). Most were conducted in the contiguous United States (81%) and some focused on Puerto Rico (15%) and the U.S. Virgin Islands (4%). Of the 13 studies on pharmacies post-EWCEs, 9 (69%) focused on hurricanes, 1 (7%) was on wildfires, and the remaining 3 (31%) focused on general disaster preparedness. Study populations for papers focusing on pharmacy deserts included residents living outside of metro-Atlanta, residents of 4 major cities (New York City, Los Angeles, Houston, and Chicago), or residents in low-income communities in Chicago.

**Table 1.** Characteristics of 26 Studies Included in the Scoping Review.

Author	Year of publication	Focus	Study location	Study design	Study population	#
Amstislavski	2012	Pharmacy deserts	Northeast	Cross sectional	New York City Residents	20
Carameli	2013	EWCEs	West	Cross sectional	Los Angeles County Residents	6
Qato	2014	Pharmacy deserts	Midwest	Cross sectional	Black/African American and Hispanic/Latino communities in Chicago	28
Arya	2016	EWCEs	Northeast	Cross sectional	Residents of NYC affected by Hurricane Sandy	17
Davidow	2016	EWCEs	Northeast	Cross sectional	Survey respondents living in New Jersey when Hurricane Sandy occurred	7
Ford	2016	EWCEs	N/A	Cross sectional	N/A	8
Pednekar	2018	Pharmacy deserts	Northeast	Cross sectional	Elderly residents of Pennsylvania enrolled in a State Pharmaceutical Assistance Program	22
Qin	2018	Pharmacy deserts	Northeast	Cross sectional	Residents of Baltimore, Maryland seeking prescription-EPT with a focus on Black/African American and Hispanic/Latino populations.	23
Barber	2019	Pharmacy deserts	Midwest	Cross sectional	Random sample of 1,003 women ages 18-19 living in a county in Michigan in 2008-2009; and 82 pharmacies in the county.	29
Jiménez-Mangual	2019	EWCEs	South; Puerto Rico	Cross sectional	Patients receiving care at 3 selected community pharmacies.	9
Johnson	2019	Pharmacy deserts	South	Cross sectional	Residents in a low-income Black/African American community	25
Lozo	2019	Pharmacy deserts	Northeast	Cross sectional	Residents of the 10 New Jersey cities included in the study	24
Smith	2019	EWCEs	South; Puerto Rico	Cross sectional	Patient populations affected by Hurricanes Harvey, Irma, Maria in Puerto Rico, Texas, and Florida.	12
Henkel	2020	EWCEs	West; Midwest; South	Cross sectional	Rural communities	14
Nair	2020	EWCEs	U.S. Virgin Islands	Cross sectional	Hurricane Maria survivors who were recipients of EPAP benefits	15
Romolt	2020	EWCEs	South; Puerto Rico	Cross sectional	N/A	10
Sharpe	2020	EWCEs	South	Cross sectional	N/A	11
Baek	2021	EWCEs	South	Cohort	Patients aged 18-105 who used a patient portal during Hurricane Harvey	5
Guadamuz a	2021	Pharmacy deserts	West; Midwest; Northeast; South	Cross sectional	Residents of the studied cities with a focus on Black/African American and Latino residents.	26
Guadamuz b	2021	Pharmacy deserts	West; Midwest; Northeast; South	Cross sectional	Residents of New York City, Los Angeles, Chicago, and Houston.	27
Neuner	2021	Pharmacy deserts	West; Midwest; Northeast; South	Cohort	Women aged ≥66 in mainland U.S. with a first diagnosis of stage 0-III breast cancer from 2011-2015.	19
Oliveira	2021	Pharmacy deserts	South	Cross sectional	Residents of Georgia	21
Rodríguez-Madera	2021	EWCEs	Puerto Rico	Cross sectional	Puerto Ricans affected by Hurricane Maria	16
Wisseh	2021	Pharmacy deserts	West	Cross sectional	Residents of Los Angeles County	3
Tseregounis	2022	EWCEs	West	Cross sectional	Patients on opioid therapy that fill at the included pharmacies.	13
Ying	2022	Pharmacy deserts	West; Midwest; Northeast; South	Cross sectional	Residents of New York City, Los Angeles, Chicago, and Houston.	18

### Pharmacies Post-EWCEs

The studies that assessed the role of pharmacies post-EWCEs (N = 13) showed the importance of pharmacists in providing care to individuals impacted by EWCEs (Table 2).^5-17^ However, these studies also revealed that many pharmacies were not equipped with disaster preparedness protocols despite the existence of statewide policies to help pharmacies lead disaster recovery efforts.^8^ This lack of disaster preparedness affected the functionality and accessibility of pharmacies in times when care was most needed.

**Table 2.** Preparedness of and Access to Pharmacies Post-EWCE.

Author	Year of publication	P/A	Main findings	EWCE	#
Carameli	2013	A	Barriers that prevented patients from accessing medications included restrictive insurance benefits, patients’ resistance to mail order, and higher copayments for increased prescription quantity.	Any	6
Davidow	2016	A	Following Hurricane Sandy, the uninsured and evacuees needed greater medical attention and were less likely to be able to fill a prescription. Only 15% of New Jerseyans were aware of the EPAP.	Hurricane	7
Arya	2016	P	Of those community pharmacies surveyed 91% were aware of EPAP, 81% found the information easy to obtain, 74% suffered from structural damage, 71% were able to reopen within 1 month, and 88% had enough staff to resume normal operations. Pharmacies did not have a clear-cut understanding of what could be done to prevent damage.	Hurricane	17
Ford	2016	P	Median number of RPHEs included in state pharmacy documents was one. Ten states incorporated language specific to public health emergency refill dispensing, and among these, only six allowed 30-day refill quantities.	Any	8
Smith	2019	P/A	P: E-prescribing activity in Puerto Rico did not return to baseline levels during the study period, and medication transactions returned to normal only after an extended period following Hurricane Maria. A: Emergency declarations issued during Hurricanes Harvey and Irma allowed pharmacists to dispense up to a thirty-day supply of a medication without a prescriber’s authorization.	Hurricane	12
Jiménez-Mangual	2019	A	77% of patients of community pharmacies surveyed reported problems related to their medications and having trouble either contacting or getting to their pharmacy following the EWCE.	Hurricane	9
Henkel	2020	P	Community pharmacies in rural areas were less likely to have emergency power ([OR]=0.59; 95% CI: 0.32-1.07).	Any	14
Nair	2020	P	Through local pharmacies enrolled with EPAP, continuity of care for the uninsured was safeguarded. EPAP services are a critical component of disaster recovery efforts to address chronic care medication needs of displaced disaster survivors.	Hurricane	15
Romolt	2020	P	Only 11.1% of pharmacies remained open in Puerto Rico three days after Hurricane Maria and pharmacy operations recovered 10 times slower, compared to pharmacy operations in Florida after Hurricane Irma which reached baseline operations less than one week following Hurricane Irma’s landfall.	Hurricane	10
Sharpe	2020	P	In the Hurricane Florence-impacted region, counties located along the coast had the most suboptimal pharmacy functionality, whereas counties located more centrally within North Carolina and South Carolina had more optimal pharmacy functionality.	Hurricane	11
Baek	2021	A	Patient portal messages during Hurricane Harvey showed that patients frequently messaged providers regarding prescription needs. Their medications were destroyed because of flooding, they struggled to get their medication because of weather-related pharmacy closures or lack of medication available at their local pharmacy and struggles with having a delayed prescription approval from a provider.	Hurricane	5
Rodríguez-Madera	2021	P	Policy makers, healthcare workers, and patients, perceived the health sector as being chaotic and lacking clear guidelines on how to provide services.	Hurricane	16
Tseregounis	2022	A	After the Camp Fire, there were significant spikes in the proportions of early fills, late fills, and increases in prescriber and pharmacy changes.	Wildfire	13

Abbreviations: A, accessibility; P, preparedness.

In 1 paper, amid dealing with EWCEs, policy makers, healthcare workers, and patients perceived the health sector as being chaotic and lacking clear guidelines on how to provide services or cope with personal crises while working under extreme conditions.^16^ In these times of disorder, pharmacists were seen as accessible and reliable healthcare professionals by other providers and patients. In 1 study, 94% of surveyed patients who received care at community pharmacies in Puerto Rico after Hurricane Maria reported that pharmacists were available to help them and 95% reported that the information provided by pharmacists was “trustworthy/very trustworthy.”^9^ When interviewing physicians, pharmacists, and insurers, a study found that pharmacists were believed to have the primary responsibility for patients’ medication continuity during an EWCE.^6^

Studies related to pharmacies post-EWCE were further categorized with regard to disparities related to their level of preparedness (N = 8) and access (N = 6).

#### Pharmacy preparedness post-EWCEs

Pharmacy preparedness varied widely depending on the location of the pharmacy and knowledge of emergency pharmacy laws and programs. Pharmacies in lower income areas were less likely to have emergency power and offsite data backup or a formal disaster plan compared with pharmacies in higher income areas.^14^ In the Hurricane Florence-impacted regions of North Carolina and South Carolina, locations along the coast had suboptimal pharmacy functionality due to a lack of preparedness, whereas counties located more centrally had higher functioning pharmacies throughout the disaster.^11^

Continuity of care for the uninsured was safeguarded with the Emergency Pharmaceutical Assistance Program (EPAP). This is a program that allows enrolled pharmacies to process claims for prescription medications, certain medical supplies, vaccinations, and some forms of medical equipment for eligible people who live in a federally identified disaster area. Enrollment in EPAP services was determined to be a critical component of disaster recovery efforts to address chronic care medication needs of displaced disaster survivors.^15^ Additionally, a study of community pharmacies in areas most severely affected by Hurricane Sandy in New York City, found that 91% were aware that EPAP allowed for prescription refill allowances based on any evidence of a previous fill to enable emergency continuous medication access, and 81% found the information easy to obtain.^17^ One study estimated pharmacy preparedness of states by looking for the presence of the 2006 Rules for Public Health Emergencies (RPHE) written by the National Association of Boards of Pharmacy (NABP) in State Boards of Pharmacies’ legal documents. It was found that the median number of RPHEs present in these documents was 1 per state board and 10 states incorporated language specific to public health emergency refill dispensing. Among these 10 states, only 6 allowed 30-day refill emergency quantities.^8^

Studies have used the time to recovery and transaction volumes to analyze the preparedness of pharmacies post-EWCEs. Two studies showed that the time to return to normal operations for pharmacies in Puerto Rico post Hurricane Maria was slower than that of pharmacies in Florida and Texas post Hurricanes Harvey and Irma.^10,12^ Only 11.1% of pharmacies remained open in Puerto Rico 3 days after Hurricane Maria. Additionally, pharmacy operations recovered slowly, at an average daily rate of 3.9% that were up and running before reaching baseline operations before the hurricane.^10^ Puerto Rico pharmacy operations after Hurricane Maria recovered 10 times slower on average compared to pharmacy operations in Florida after Hurricane Irma which reached baseline operations less than 1 week following Hurricane Irma’s landfall.^10^

Although e-prescribing and dispensing of medications decreased considerably during each hurricane, transaction volumes returned to normal levels in the days immediately following Hurricanes Harvey and Irma in Texas and Florida. However, e-prescribing activity in Puerto Rico did not return to baseline levels during the study period, and medication transactions returned to normal only after an extended period following Hurricane Maria.^12^ A study interviewing 52 community pharmacies in areas severely affected by Hurricane Sandy in New York City, found that issues other than power outages were more important contributors to a pharmacy’s return to normal operational status.^17^ As Hurricane Sandy approached the coast, pharmacies did not have a clear understanding of what could be done to prevent damage.^17^ Of those pharmacies surveyed, 74% suffered from structural damage and 71% were able to reopen within 1 month. Despite staffing challenges, 88% of pharmacies had enough pharmacists and staff to resume normal operations.^16^ After the storm, most pharmacies had to replace medications, a some had to replace equipment, and a few had to completely renovate their stores.^17^

#### Access to pharmacies post-EWCEs

Studies have shown that issues with accessing pharmacies and medications post-EWCEs are often due to pharmacy closures, inability to contact prescribers, and insurance challenges.^5-7,9,12,13^ In 1 paper, barriers that prevented patients from accessing medications included restrictive insurance benefits, patients’ resistance to mail order, and higher copayments for larger prescription units.^6^ A study interviewed patients of community pharmacies in Puerto Rico after Hurricane Maria to assess patients’ medication needs and level of satisfaction with community pharmacy services. The majority (77%) of respondents reported problems related to their medications and 48% reported having trouble either contacting or getting to their pharmacy following the hurricane.^9^ In addition to these barriers, a study post-Hurricane Sandy determined that only 15% of New Jerseyans were aware of the EPAP.^7^ Following Hurricane Sandy, uninsured persons and evacuees needed greater medical attention, experienced the greatest hurricane impact, and were less likely to be able to fill a prescription.^7^ Furthermore, a study determined that California wildfires were associated with disrupted access to prescription opioids for patients receiving long-term opioid medications.^13^ Among patients on chronic pain management, the study noted that there were significant spikes in the proportions of early fills and late fills of their medications, as well as an increase in prescriber and pharmacy changes.^13^

Patient portal messages during Hurricane Harvey showed that patients frequently messaged providers regarding prescription needs due to their inability to acquire their medications and limited access to pharmacies.^5^ Patients described situations in which their medications were destroyed or unreachable because of flooding and requested a prescription refill.^5^ Additionally, some patients struggled to get their medication because of weather-related pharmacy closures or lack of medications available at their local pharmacy.^5^ Messages from patients often included context related to disruption of care due to difficulties in acquiring medications from a pharmacy or a delayed prescription approval from a provider during the hurricane.^5^ To alleviate issues like this, emergency federal declarations issued during Hurricanes Harvey and Irma granted pharmacists the authority to dispense up to a 30 day supply of a patient’s medications without a prescriber’s authorization.^12^

### Pharmacy Deserts

Studies related to pharmacy deserts (N = 13) addressed their prevalence and the factors affecting pharmacy deserts (Table 3). One study focusing on identifying all potential pharmacy deserts in Los Angeles County, found that 25% of the 2323 county census tracts were pharmacy deserts.^3^ Another study that mapped out pharmacy deserts in New York City, Los Angeles, Houston, and Chicago found that of 4654 neighborhoods identified in these 4 cities, 670 (14%) were located in pharmacy deserts based on their distance to the nearest pharmacy.^18^ Studies related to pharmacy deserts can be further categorized based on their geographic location (rural, suburban, and urban; N = 5), level of income (N = 3), and race and ethnicity (N = 7).

**Table 3.** Disparities With Regard to Pharmacy Deserts.

Author	Year of publication	G/I/R	Main findings	#
Amstislavski	2012	G/I	G: Residents in the “outer” boroughs of New York City had the least geographic access to chain pharmacies at only 0.06 (±0.13) pharmacies per 1000 residents, as compared to residents living in Manhattan, which had 0.43 (±0.30) pharmacies per 1000 residents. I: Independent pharmacies are more likely to be in low-income neighborhoods while chain pharmacies are more likely to be in lower poverty areas of NYC. Chain pharmacies have larger facilities and a wider range of prescription medications in stock than independent pharmacies, offer more preventive health services, and are more likely to have broader hours.	20
Qato	2014	R	The number of pharmacies was lower in segregated minority communities than in segregated white communities and integrated communities. There were disproportionately more pharmacy deserts in segregated Black communities.	28
Pednekar	2018	G	Pharmacy deserts had fewer chain and independent pharmacies and less delivery and 24-hour services. Pharmacy deserts were more prominent in rural areas of Pennsylvania.	22
Qin	2019	G	Limited geographic access to pharmacies may prevent utilization of EPT.	23
Lozo	2019	I	An increase in median household income indicated more pharmacy naloxone availability compared to neighborhoods with a lower median household income.	24
Barber	2019	R	Pharmacy deserts are more prevalent in Black/African American and Hispanic/Latino neighborhoods and tend to be independent pharmacies open fewer hours, have fewer female pharmacists, more difficult access to condoms (49% on the shelf vs 85% behind glass, behind the counter, or not available), and fewer self-check-out options (3% vs 9%). Relative to white women, Black/African American women may face a “contraception desert.”	29
Johnson	2019	I/R	Residents of low-income community within Nashville felt as if they were treated more negatively because of where they live, the insurance card they present, and assumptions made about them. Black/African American patients felt they were being treated differently and experienced negative stereotyping at their pharmacies resulting in feelings of disconnectedness between patients and pharmacists.	25
Neuner	2021	G	Among 45 722 patients with breast cancer living in the United States, more than 11% lived in census tracts where no pharmacy was within recommended driving distances. Vaccination in these patients was less likely (adjusted OR 0.92 [95% CI 0.86-0.96]).	19
Guadamuz a	2021	R	Within the 30 most populous US cities, there are persistently fewer pharmacies located in Black/African American and Hispanic/Latino neighborhoods than White or diverse neighborhoods between 2007 and 2015.	26
Guadamuz b	2021	R	As of 2020, pharmacies in Black/African American and Hispanic/Latino neighborhoods were more likely to close and less likely to offer immunization, 24-hour, and drive-through services than pharmacies in other neighborhoods	27
Oliveira 2021	2021	G	Counties and census tracts with lower populations tended to have fewer pharmacies. Large urban areas have more access to pharmacies than rural areas and small cities in Georgia.	21
Wisseh	2021	R	Pharmacy deserts in LA County have a denser population, more renters, more residents that speak English as a second language, more low income residents, and more Black/African American and Hispanic/Latino residents.	3
Ying	2022	R	Of 670 total pharmacy deserts, only 29 (2.2%) were in predominantly white neighborhoods and Latino, Black, and diverse neighborhoods had 344 (25.9%), 203 (25.0%), and 94 (8.0%) pharmacy deserts, respectively. Predominantly Black neighborhoods accounted for 7.3% of total pharmacy deserts based on travel time by car, then Latino (4.4%), diverse (0.9%), and white (0.2%) neighborhoods	18

Abbreviations: G, geographic area; I, income; R, race/ethnicity.

#### Geographic area

Studies have shown that there is a relationship between rural counties and a lower number of pharmacies compared to urban counties and a higher number of pharmacies.^19-22^ A study investigating the spatial equity of pharmacies in Georgia found that counties and census tracts with lower populations and lower population densities tended to have fewer pharmacies compared to counties and census tracts with higher populations and higher population densities.^20^ Their results indicated that there is a spatial inequity of pharmacy distribution in the state, and areas outside of Metro-Atlanta have a high level of pharmacy deserts, especially in small cities and rural areas.^20^ A study analyzing pharmacy deserts in Pennsylvania found that pharmacy deserts had significantly fewer chain and independent pharmacies, less medication delivery, and fewer 24-hour services in pharmacies as compared to areas without pharmacy deserts.^21^ Pharmacy deserts were also more prominent in southcentral, northwest, and northeast regions of the state which represent rural areas.^21^

Disparities in pharmacy access are not limited to rural versus urban areas, when comparing medication access among neighborhoods in New York City. A study found that residents in the “outer” boroughs of New York City (Bronx, Brooklyn, Queens, and Staten Island) had the least geographic access to chain pharmacies at only 0.06 (±0.13) pharmacies per 1000 residents, as compared to residents living in Manhattan, which had 0.43 (±0.30) pharmacies per 1000 residents.^19^

Pharmacy deserts have larger implications than reduced medication access, the important services they provide, such as vaccination and expedited partner therapy (EPT), have become sparser as well. A study determining barriers to implementing expedited partner therapy for sexually transmitted diseases in Baltimore, Maryland found that limited geographic access to pharmacies may prevent effective utilization of EPT.^22^ Another study examined whether pharmacy access was associated with decreased influenza vaccination in patients recently diagnosed with breast cancer in mainland United States. In the 457 522 patients examined in this study, over 11% of breast cancer patients lived in census tracts where no pharmacies were within recommended driving distances from the population-weighted tract center. This resulted in decreased rates of vaccination in the year after diagnosis for breast cancer patients living in these pharmacy deserts.^23^

#### Level of income

Lower income neighborhoods have been linked to fewer pharmacies, especially chain pharmacies, compared to higher income neighborhoods. One study found that independent pharmacies were more likely to be in the low-income neighborhoods of New York City while chain pharmacies were more likely to be located in higher income areas.^19^ Chain pharmacies typically have larger facilities and have a wider range of prescription medications in stock than independent pharmacies and offer a broader range of preventative health services, including vaccinations and self-service blood pressure monitoring.^19^ They are also more likely to remain open and to have a pharmacist in the store to dispense prescription medications at night, on the weekends, and holidays.^19^

A study investigating the availability of naloxone at pharmacies in cities across New Jersey found that an increase in median household income indicated more pharmacy naloxone availability compared to neighborhoods with a lower median household income.^24^ Along with having limited access to pharmacies, another study interviewed residents living in a low-income community within Nashville, Tennessee on their perceptions of pharmacy. They stated that they felt as if they were treated more negatively because of where they lived and the insurance card they presented and that unsupported assumptions were made about them by pharmacy employees.^25^ As a result, residents living in these lower income communities had limited access to pharmacies and were also more likely to experience stigma when seeking care.

#### Race and ethnicity

Pharmacy deserts were predominantly found in Black/African American and Hispanic/Latino neighborhoods. A study examining the availability of pharmacies within the 30 most populous US cities between the years 2007 to 2015 found persistently fewer pharmacies located in Black/African American and Hispanic/Latino neighborhoods than White or diverse neighborhoods.^26^ As of 2020, pharmacies in Black/African American and Hispanic/Latino neighborhoods were also more likely to close and less likely to offer immunization, 24-hour services, and drive-through services than pharmacies in other neighborhoods.^27^ Throughout the period 2000 to 2012, a study examined whether trends in the availability of pharmacies varied across communities in Chicago with different racial or ethnic compositions.^28^ The number of pharmacies was lower in segregated minority communities than in segregated white communities and integrated communities and there were disproportionately more pharmacy deserts in segregated Black/African American communities.^28^ Pharmacy deserts in LA County had a denser population, more renters, more residents that spoke English as a second language, less vehicle ownership, more residents living under the federal poverty level, more Black/African American and Hispanic/Latino residents, more areas with higher crime against property and people, and fewer health professionals to serve the area.^17^

In addition to pharmacy deserts being more prevalent in communities with persons of color, patients in these communities report experiencing discrimination leading to additional disparities.^25,29^ For example, relative to white women, Black/African American women noted “contraception desert,” wherein they lived closer to pharmacies but those pharmacies had characteristics that impeded the purchase of contraception.^29^ The study found that pharmacies in their neighborhoods tended to be independent pharmacies that were open fewer hours per week, and have fewer female pharmacists, more difficult access to condoms (49% on the shelf vs 85% behind glass, behind the counter, or not available), and fewer self-check-out options (3% vs 9%).^29^ Qualitative interviews from another study revealed that Black/African American patients felt they were being treated differently because of their low-income, residential neighborhood and experienced negative stereotyping at their pharmacies resulting in feelings of disconnectedness between patients and pharmacists.^25^

## Discussion

In this scoping review we examined pharmacy preparedness and accessibility post-EWCEs, as well as disparities within pharmacy deserts. These topics were reviewed together to assess how ready pharmacies are for an EWCE and if 1 occurs, what populations are most vulnerable. We found that although community pharmacies were highly accessible healthcare locations amid EWCEs, they were not readily prepared to help safeguard the pharmacy and continue delivery of patients’ medications, with pharmacies in lower income and rural communities being least prepared.^7,11,14^ Finally, most reviewed studies indicated that pharmacy deserts were more prevalent in rural, low-income, or Black/African American and Latino neighborhoods.^3,18-29^ As such, communities living in these areas were at an increased risk of adverse outcomes given that they were disproportionately affected by both pharmacy deserts and pharmacies not being adequately prepared for EWCEs.

Past literature reviews have exclusively defined the role of community pharmacists amid disaster response and emergencies^30,31^; therefore, our paper adds to the literature by reviewing the overall preparedness and accessibility of pharmacies and pharmacists post-EWCEs and pharmacy deserts. Although, pharmacists were perceived as accessible, reliable, and trustworthy providers and having the primary responsibility for the continuity of patient’s medications, they were not adequately prepared to assure the continued delivery of care amid EWCEs.^6,9,16^ Consequently, access to pharmacies was limited in times of EWCEs which resulted in patients losing access to critically needed medications. Common barriers patients faced included shortages of medications at their pharmacy, pharmacy closures, an increased cost of refills, and not being able to get prescriptions from their providers.^5^ One study highlighted an increase in prescriber and pharmacy changes after a wildfire, indicating that patients had to find alternative persons and locations from which to obtain their medications as an additional stressor of the EWCE.^13^ By exploring different avenues to address these barriers ahead of time, pharmacists can minimize disruptions patients face with their medications. There may not be a list of readily available solutions for pharmacies looking to support patients through EWCE. Actions may be highly individualized based on pharmacy characteristics, anticipated needs of the local population, and availability of and interlinkages with nearby health systems and other pharmacies.

Although we did not identify any studies that examined specific interventions to mitigate these challenges, we believe that states and pharmacies more prone to EWCEs should use global resources available to them such as the NABP published emergency response guidelines, to help create disaster preparedness protocols. The inclusion of programs like EPAP into emergency protocols and alternative avenues of communication between patients and providers should be explored further to mitigate challenges patients face. In some cases, there may be some lead time (on the order of hours to days) before more predictable ECWEs occur, for example in the case of hurricanes or blizzards, during which emergency declarations can be implemented in advance allowing pharmacists a head start in preparing services and dispensing necessary medications. Additionally, to prevent medication shortages for these, pharmacists can order extra supplies of commonly dispensed medications to their pharmacies and can proactively reach out to patients to refill medications on time or a few days in advance of due dates. Although pharmacists were aware of the EPAP which offers a free 30-day refill of prescription medications, few patients had knowledge of it.^7^ Information about programs like EPAP should be distributed widely so patients are aware of these helpful resources during EWCEs. Lastly, state board of pharmacies can implement mandatory continued education courses to ensure pharmacists are well-versed in emergency response. Puerto Rico, which has a history of delayed recovery should be researched further for potential factors which may include its healthcare infrastructure, geography, or relevant laws and regulations.^10,12^ Disaster planning of pharmacies must be studied further to develop emergency response protocols that pharmacies can implement in case of future EWCEs.

Pharmacy deserts are found across the US, with residents living in rural, lower income, and Black/African American and Hispanic/Latino neighborhoods being affected the most.^3,18-29^ These communities are also less likely to have access to chain pharmacies.^19,27,29^ This is an important finding because in comparison to independent pharmacies, chain pharmacies have longer hours, offer broader services, and are more likely to have a wider range of medications in stock.^19,27,29^ As a result, studies have found pharmacy deserts to be a reason for decreased rates of flu vaccination among certain populations, reduced use of EPT, and lower availability of naloxone among residents living in rural, lower income, and Black/African American and Hispanic/Latino neighborhoods.^22-24^ Future research should address how equity can be enhanced for residents living in pharmacy deserts to increase their access to pharmacies and pharmacy offered services.

Finally, residents living in rural areas, or urban areas with high density, low income, or racial diversity are most vulnerable to an EWCE due to a presence of pharmacy deserts. Pharmacies that do exist in these areas are less likely to be prepared for EWCEs, making residents particularly vulnerable to losing medication access.^7,11,14^ Pharmacy deserts overlapping with areas prone to EWCEs should be further investigated to identify the pharmacies and communities that are at highest risk. Proactive outreach to better prepare pharmacies for an EWCE, providing incentives to pharmacists to work in underserved areas, and training of pharmacists on the needs of communities in pharmacy deserts can be implemented. Patients living in those pharmacy deserts should be educated to have their own medication preparedness plan.

There are some limitations to our review. We included studies written in English within the last 10 years from the following online databases: PubMed, Embase, and Web of Science. As such, there may have been other relevant studies and abstracts in other databases or written in other languages that were not included. Nearly all included studies were cross-sectional. Future research should include longitudinal studies which may shed light on speed and efficiency of recovery efforts of pharmacies post-EWCEs. Most included studies were about pharmacies post hurricanes and wildfires. As climate change progresses, we will continue to experience a rise in other EWCEs necessitating more research related to their implications on the delivery of care.^32^ Further research should examine how pharmacy deserts affect patients’ access to treatment and preventative services offered in pharmacies particularly in rural, low income, and Black/African American and Hispanic/Latino neighborhoods.

To help prepare and lead recovery efforts amongst communities and pharmacies more prone to EWCEs, it is imperative that measures are taken to keep pharmacists informed and pharmacies prepared ahead of time. To help achieve health equity, the implications of pharmacy deserts among minority and low-income communities should be further examined and interventions should be conceived to improve access to pharmacists and pharmacy services. The development and implementation of pharmacy disaster protocols, particularly among communities in pharmacy deserts, should be studied further to enhance the continued delivery of care and minimize harm to patients post-EWCEs.
